# Exercise Rehabilitation for Heart Failure and Associated Cardiomyopathies

**DOI:** 10.1007/s11886-025-02313-9

**Published:** 2025-12-02

**Authors:** Macy E. Stahl, Nathan R. Weeldreyer, James P. MacNamara, Patricia F. Rodriguez Lozano, Jason D. Allen

**Affiliations:** 1 Department of Kinesiology, School of Education and Human Development, University of Virginia, Charlottesville, VA 22903 USA; 2 Faculty of Nursing, College of Health Sciences, University of Alberta, Edmonton, AB Canada; 3 Department of Radiology and Medical Imaging, University of Virginia Health, Charlottesville, VA USA; 4 Department of Medicine, University of Virginia School of Medicine, Charlottesville, VA 22904 USA

**Keywords:** Heart failure, Exercise, Cardiac rehabilitation, HFrEF, HFpEF, HfmrEF, Hypertrophic cardiomyopathy, ANOCA

## Abstract

**Purpose of Review:**

To summarize and provide insight into the role of exercise rehabilitation for heart failure, cardiomyopathies and associated conditions. We provide an overview of the evolution of exercise training from “bed rest” to current guidelines and highlight emerging approaches.

**Recent Findings:**

Exercise training appears to be safe and provide benefits for patients with heart failure. Emerging evidence also suggests potential benefit in hypertrophic cardiomyopathy as well as angina with non-obstructive coronary arteries, though more data are needed for widespread implementation.

**Summary:**

Given appropriate precautions and clinical assessments, exercise is a unifying therapy across most heart failure conditions regardless of ejection fraction. Exercise training appears to be safe, and beneficial in terms of improvements in functional capacity and health-related quality of life. Larger controlled trials are needed to better examine the impact of exercise on hard clinical endpoints such as HF hospitalizations (likely beneficial) and cardiovascular mortality.

## Introduction

Heart Failure (HF) is a major health concern and is the leading cause of hospitalization among aging Americans, with estimated direct and indirect costs of treatment at ~$39.2 billion [1] and a ~ 75% 5-year mortality rate [2]. Approximately 80% of patients are over 60 years old [3]. HF manifests as the inability of the heart to adequately meet the metabolic demands of the body, which is especially evident during physical activity, when metabolic demands are increased. Patients with HF suffer from shortness of breath, fatigue and exercise intolerance [4]. Improving VO_2peak_ is an important clinical goal in HF as it is correlated with reduced mortality rate and increased quality of life (QoL) [5, 6]. To this end, exercise rehabilitation is now considered a class IA recommendation for people with HF [7], with guidelines recommending moderate-intensity aerobic modalities [4, 8], often in conjunction with resistance training [8–10].

There are two main sub-type classifications in the diagnosis of HF, which relies heavily on the assessment of left ventricular function, specifically its ejection fraction (EF). Heart failure with reduced ejection fraction (HFrEF) defined as EF *≤* 40%, is often described as “classic heart failure,” is well characterized, and effective therapies for patients are available [11]. Heart failure with preserved ejection fraction (HFpEF) has previously been defined with a variety of EF’s but more recently defined as *≥* 50%. Pharmacological interventions such as sodium-glucose cotransporter 2 (SGLT2) inhibitors, glucagon like peptide-1 (GLP-1)/gastric inhibitory peptide (GIP) agonists, and finerenone reduce heart failure hospitalizations in HFpEF, but their influence on pathophysiology is still being determined [12–14].

Currently, the Centers for Medicare and Medicaid Services cover exercise rehabilitation for stable patients with HFrEF but not HFpEF or other forms of cardiomyopathies [15, 16]. To provide insight into the reasoning for this and potential new approaches in the continuing evolution of exercise rehabilitation for HF it is worthwhile to review how we arrived at the current guidelines.

## A Brief History of Exercise Training in Heart Failure (Outlined in Fig. 1)

As recently as 1965, bed rest was standard of care for treatment post myocardial infarction. “*Prolonged complete bed rest in conjunction with conventional forms of therapy is being utilized in patients with idiopathic myocardial disease and cardiac dilatation in an effort to unload the heart*.” [17] This dogma was challenged in 1968 by Hellerstein and collegues who published data showing “*that an active*,* supervised*,* conditioning program can be used safely in the treatment of selected subjects with coronary heart disease*,* infarction*,* and angina*,* but not in congestive failure.”* [18].

**Fig. 1:**
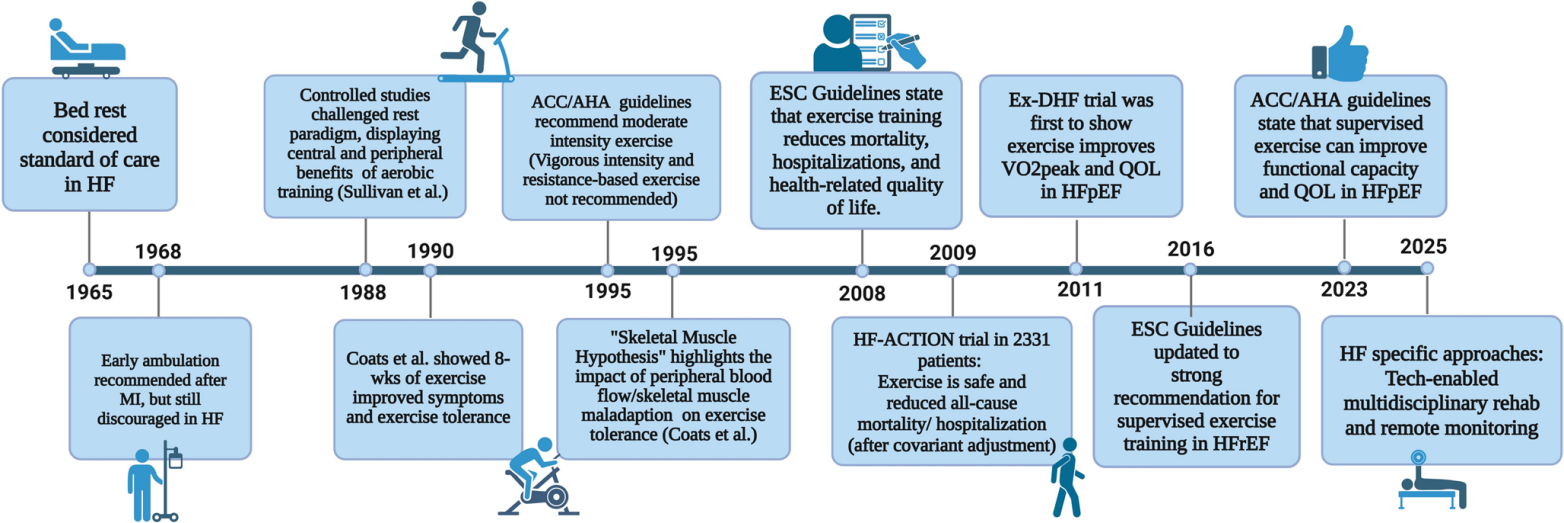
A Brief History of Exercise Training in Heart Failure. Created in BioRender. Stahl, M. (2025) https://BioRender.com/gw8trbl

### a) Heart Failure with Reduced Ejection Fraction (HFrEF)

In the late 1980’s Cobb and colleagues showed, in a series of elegant physiological studies, that exercise participation for patients with left ventricular systolic dysfunction had beneficial effects on submaximal, as well as maximal, exercise performance and involved peripheral adaptations [19, 20]. Similarly, in 1990, Coats demonstrated improved exercise capacity and reduced symptoms in patients with chronic heart failure (CHF) following physical training programs, stating: *“The commonly held belief that rest is the mainstay of treatment of chronic heart failure should no longer be accepted.”* [21].

Accordingly, the 1996 American College of Cardiology (ACC)/American Heart Association (AHA) Guidelines reflected these new developments by recommending in HFrEF, that *“Intense physical exertion should be discouraged*,* but moderate exercise to tolerance should be strongly encouraged”.* At this point resistance-based activities such as, push-ups or weightlifting, were discouraged due to “*a fear of acute afterload stress to the left ventricle”* [22]. Coats again challenged the paradigm with publication of the Skeletal Muscle Hypothesis of CHF, underscoring that importance of maladaptation in peripheral blood flow and skeletal muscle that limit exercise capacity and initiate an *“abnormal reflex cardiopulmonary control which may also be harmful in the progression of the syndrome”* and suggesting “t*he judicious use of exercise training of peripheral muscles in CHF”* [23].

By 2008 the European Society of Cardiology (ESC) Guidelines reviewed evidence from several systematic reviews and meta-analyses of small studies and concluded that *“physical conditioning by exercise training reduces mortality and hospitalization when compared with usual care alone*,* and improves exercise tolerance and health-related quality of life”* and that “*Cardiac rehabilitation programmes following a cardiovascular event or episode of decompensation represent an effective treatment option for patients with HF.”* [24] The following year, the outcomes of the landmark HF-ACTION trial involving 2331 patients with HFrEF showed that exercise in patients with HFrEF was safe and had a modest (non-statistically significant) reduction in all-cause mortality or all-cause hospitalization [25]. After adjusting for heart failure etiology and high prognostic covariates independent of treatment assignment (duration of CPX test, LVEF, Beck Depression Inventory II score, history of atrial fibrillation or flutter), exercise training was found to reduce the incidence of all-cause mortality or all-cause hospitalization by 11% (*p* = 0.03). Subsequently, in 2014 the Centers for Medicare and Medicaid Services provided coverage for exercise training and cardiac rehabilitation in stable HFrEF, and the 2016 ESC Guidelines included “*strong*” recommendations for supervised exercise training in patients with HFrEF [4].

### b) Heart Failure with Preserved Ejection Fraction (HFpEF)

To date, the data from exercise studies in patients with HFpEF is relatively sparse compared to HFrEF, with a lack of large-scale randomized controlled trials [26]. The majority of exercise training trials in HFpEF have been smaller single center trials, which weakens the strength of evidence, despite consistently demonstrated improvements in VO_2peak_ [27–37]. The 2011 Exercise training in Diastolic Heart Failure (Ex-DHF) pilot trial (*n* = 64), suggested that 3 months of supervised endurance/resistance training improved VO_2peak_ and QoL [36]. Overall, meta-analyses suggest mean increases of 1.66–2.8 ml/kg/min following supervised exercise training in HFpEF [26, 38–41]. Assuming a starting VO_2peak_ of ~ 16 ml/kg/min this would result in a 10–17% increase in cardiorespiratory fitness, a clinically meaningful change associated with improved outcomes [26].

In the last 5 years, two large scale multicenter trials have been published examining the effect of exercise in patients with HFpEF. In 2021, the OptimEx-Clin study compared 3 months of high-intensity interval training (HIIT) vs. moderate continuous training (MCT) versus usual care (UC). HIIT and MCT increased VO_2peak_ by 1.1 and 1.7 ml/kg/min respectively, which was statistically significantly greater than UC, which declined by 0.6 ml/kg/min. However, neither exercise group met the prespecified minimal clinically important difference (+ 2.5 mL/kg/min) compared with the UC [29].

The 2025 Ex-DHF study is the largest exercise trial to date in patients with HFpEF, with 322 patients included in the primary analysis [42]. Patients were randomized to either UC or a combination of aerobic and resistance training. They found no effect at 12-months on the primary endpoint of a modified clinical outcome score, nor on secondary endpoints of all-cause mortality or number of HF hospitalization. Interestingly, patients randomized to exercise training increased their VO_2peak_ and NYHA class *(p* < 0.001), with adherence to exercise training associated with better results of the modified Packer score.

Furthermore, a growing body of evidence suggests that coronary microvascular dysfunction (CMD) plays a mechanistic role in a significant subset of HFpEF patients. This is particularly in females presenting with angina and nonobstructive coronary arteries (ANOCA) [43] (see below).

Although Medicare does not currently cover cardiac rehabilitation for HFpEF, there is growing recognition of its potential benefits. A joint scientific statement from the AHA and ACC in 2023 highlighted that supervised exercise therapy can improve exercise capacity and QoL in individuals with HFpEF, sometimes more effectively than medications [26].

### c) The Current Data 

The 2024 Cochrane Intervention review of exercise-based cardiac rehabilitation for adults with HF provides a comprehensive analysis of the data (up to December 2021). It includes 60 randomized controlled trials (8,728 participants) with a minimum of six-months follow up versus a no-exercise control [44]. The mean age of participants across included trials ranged from 51 to 81 years and were predominantly male (median 78%). The HFrEF; HF-Action [25] and The Telerehabilitation in Heart Failure Patients (TELELREH-HF) [45] made up approximately 27% and 13% of the subjects respectively. The total number of patients with HFpEF is unclear as nine trials included an undefined proportion of people with HFpEF. Participants were predominantly NYHA class II and III. The results suggest that exercise rehabilitation, compared with no-exercise control, likely improves health-related QoL and reduces the risk of hospital admissions from any cause and specifically due to HF but has no effect on risk of all-cause mortality in the short term (up to 12 months’ follow up). These effects appear to be consistent regardless of mode of delivery (medical center or home-based), type of training (just aerobic or aerobic plus resistance), or amount of exercise. A small number of trial-based economic evaluations support the acceptable cost-effectiveness of exercise based cardiac rehabilitation compared to control.

The Cochrane systematic review is also informative for this report as it identifies several areas in exercise and HF literature that are currently under-represented and worthy of attention in subsequent sections. These include females, older individuals with HF, those with mid-range ejection fraction (HFmrEF), and Individuals with hypertrophic cardiomyopathy (HCM). In addition, associated conditions that contribute to symptom burden and reduced exercise tolerance, such as CMD and ANOCA, are emerging as important therapeutic targets. We will also briefly examine new approaches to exercise training in HF including high intensity interval training (HIIT) and programs focused on the peripheral skeletal muscle tissues.

## Other HF Populations

### HF with mid-range Ejection Fraction (HFmrEF)

Left ventricular EF measured by echocardiography is the most used metric for clinical diagnosis, characterization, prognosis and treatment selection with HFrEF defined as EF *≤* 40% and HFpEF as *≥* 50%. However, as a continuous variable with a normal distribution within the population, the threshold value to define ‘reduced’ vs. “normal” vs. “preserved” EF is arbitrary [46]. In 2016 the ESC HF guidelines introduced a separate entity, HF with mid-range EF (HFmrEF; defined as EF 40–49%), to foster research in this EF range [47]. Data now suggest that HFmrEF has some intermediate features between HFrEF and HFpEF but also distinct similarities with HFrEF that warrant the term HF with ‘mildly reduced’ EF [46, 48–50].

Overall, the strength of the evidence suggests patients with a mildly reduced EF should be given the benefit of the doubt and considered for treatment with established therapies in HF with more severely reduced EF, and that future clinical trials for HF with reduced EF may consider enrolling patients with EF up to the normal range [51]. This approach is most likely a worthwhile consideration for exercise rehabilitation study inclusion criteria moving forward.

### Hypertrophic Cardiomyopathy (HCM)

HCM is a common genetic cardiomyopathy characterized by abnormal hypertrophy of the left ventricle, often accompanied by symptoms including dyspnea, angina, syncope and rarely ventricular arrhythmias [52]. Patients are typically at a heightened risk for sudden cardiac death (SCD) [53]. The prevalence of HCM is estimated to be 1:200 to 1:500 though most patients remain undiagnosed, and significant heterogeneity exits between patients in disease etiology and symptoms [54, 55]. Due to early data displaying HCM as a common cause of SCD cases in athletes during competition [56], starting in the mid-1980s individuals with HCM were historically advised to take a conservative approach to activity by limiting exercise intensity to “low intensity activities,” such as yoga and golf [57, 58]. There is now more recognition, however, that the risk of exercise-induced arrythmias or SCD in HCM are lower than once thought, and that the known benefits of exercise outweigh these risks in most patients with HCM [59–61].

Two landmark studies: the Randomized Exploratory Study of Exercise Training in Hypertrophic Cardiomyopathy (RESET-HCM), and Lifestyle and Exercise in Hypertrophic Cardiomyopathy (LIVE-HCM) evaluated the efficacy and safety of exercise in HCM. RESET-HCM found that 16-weeks of moderate intensity exercise (*n* = 136) improved fitness, and did not result in sustained ventricular arrythmias or SCD, though the power to assess safety was limited [59]. LIVE-HCM was a large, prospective cohort study (*n* = 1,660) that demonstrated vigorous-intensity exercise was equally as safe as moderate intensity exercise and sedentary behavior [60]. Thus far, moderate intensity exercise has improved VO_2peak_ by 1.3–1.8 ml/kg/min while high intensity exercise improved VO_2peak_ by 1.9 ml/kg/min [59, 62, 63]. In a small study directly comparing the two intensities, high intensity interval training was not superior to moderate intensity [64]. Furthermore, the adaptations to exercise training are not clear with one study showing improvement in peak cardiac output and another showing peripheral adaptations to training [62, 64]. Further work is needed to determine why high intensity training has such a modest response in HCM and how best exercise prescriptions can be personalized to the individual.

With this new prospective data, clinical advice has shifted to encouraging physical activity as part of a comprehensive treatment plan for patients with HCM, and this was reflected in the 2020 and 2024 AHA/ACC guidelines that support recreational exercise as a Class I recommendation for HCM patients [65]. While prospective studies in HCM have been encouraging, there is still uncertainty surrounding high-risk individuals, and certain subgroups have been excluded from previous HCM trials including those with very high resting gradients or a prior history of exercise induced syncope. Therefore, an expert evaluation and shared decision-making process is indicated in athletes and patients who wish to adopt a vigorous exercise program.

### Other Cardiomyopathies

For individuals with Arrhythmogenic Right Ventricular Cardiomyopathy, the current 2023 ESC Guidelines recommend *against* participation in high-intensity exercise and competitive sport based on data suggesting an association between high-intensity exercise and disease progression in combination with positive clinical outcomes following exercise restriction [66]. Due to limited evidence, exercise in other cardiomyopathies including dilated cardiomyopathy (DCM), and other inherited cardiomyopathies, should be based on multi-parametric risk assessment. In individuals with DCM, sports participation and vigorous exercise can be considered, but risk of progression or sudden arrest is unknown [67]. Limited evidence has suggested benefits for moderate intensity exercise in optimally managed patients with DCM [68].

For genotype positive/phenotype negative individuals, recommendations become more nuanced. For those without high-risk variants, low-to-moderate intensity exercise at the AHA recommended volume of 150 min/week is likely safe though final recommendations and decisions regarding high-intensity exercise should be based on an individualized risk assessment and periodical evaluation [69]. For those with high-risk pathogenic variants (ex: lamin A/C, *THEM43*) caution should be extended as exercise may contribute to life-threatening arrythmias [70, 71].

### Angina with Non-Obstructive Coronary Artery Disease (ANOCA)

Many individuals undergoing coronary angiography for angina and suspected ischemia present with no observable obstructive disease (ANOCA) [72]. ANOCA predominantly affects females, and patients demonstrate impaired myocardial perfusion, diastolic dysfunction, and exercise intolerance despite preserved ejection fraction. The coronary microvascular dysfunction (CMD)-HFpEF endotype is linked to endothelial dysfunction, subendocardial ischemia, and chronic inflammation [73].

Though large-scale trials are lacking, preliminary studies suggest that structured aerobic and resistance exercise may show improvements in anginal symptoms, VO_2peak_, QoL, and coronary flow reserve following moderate intensity exercise interventions [74–76]. Including CMD-HFpEF and ANOCA in the scope of rehabilitation may expand the reach and relevance of exercise programs in modern HF care.

## Other Considerations for Exercise in HF

### Age

A key limitation of the class IA recommendation for exercise training in individuals with HF [7], is that they arise largely from data involving a patient cohort sometimes two decades younger (range 51–81 years) than the median age of diagnosis for HF (77 years) [77, 78]. Very few exercise trials have achieved a mean age of participants >65 years [79]. For example, participants in HF-Action [25] and TELELREH-HF [45] had a mean age of 59 and 62 years respectively. Considering that older adults with HF experience a high prevalence of comorbidities, impaired functional capacity, reduced muscle mass and strength, and a 5-year survival of 25% [80–85], it is unclear whether the current exercise guidelines of moderate-intensity aerobic modalities [4, 8], often in conjunction with resistance training [8–10] can be tolerated by and generate functional benefits in a majority real-world HF patients. This discrepancy may contribute to the low enrolment in exercise rehabilitation for this cohort [78], and promoting the inclusion of older patients, who represent the ‘typical’ patient with HF, in to clinical trials should be a priority for future studies [78, 86]. 

### Biological Sex

The most notable sex-difference in HF is the higher incidence of ischemic heart disease and HFrEF in males, while females have greater incidence of HFpEF [87]. In terms of exercise capacity, females with HF exhibit an approximately 2.1 ml/kg/min lower VO_2peak_ than males, and show lower improvements in VO_2peak_ despite completing similar numbers of exercise sessions in cardiac rehabilitation programs [88]. Reduced exercise capacity by sex may also be evident in HCM. A recent study suggested a 7.35 ml/kg/min lower exercise capacity in females, which appeared to be associated with abnormalities in passive diastolic properties [89]. Given a VO_2_ threshold of approximately 12–15 ml/kg/min is required to perform tasks of daily living, these lower values could have consequences for functional independence. Exercise may need to be tailored differently for females to provide a more robust stimulus to facilitate the same improvements in functional capacity [90–92]. Conversely, a sub analysis of HF-Action did not display sex differences in VO_2peak_ change following 3-months of exercise training, and suggested females may benefit from a larger reduction in all-cause mortality [93]. More highly controlled studies enrolling a larger cohort of females and evaluating sex-specific impacts of exercise in patients with HF are likely required to clarify these discrepancies and inform guidelines.

## Skeletal Muscle Hypothesis Revisited. A Focus on the Peripheral Tissues

It was historically assumed that the low VO_2peak_ in HF was primarily due to the concomitant reduction in cardiac output (Q̇). However, impairments in multiple components of the Fick equation occur in HF. These include the reduced capacity to pump blood and lower skeletal muscle perfusion (convective transport) to reduced muscle mass, metabolism and oxygen extraction (diffusive transport) [23, 94–97].

**Fig. 2:**
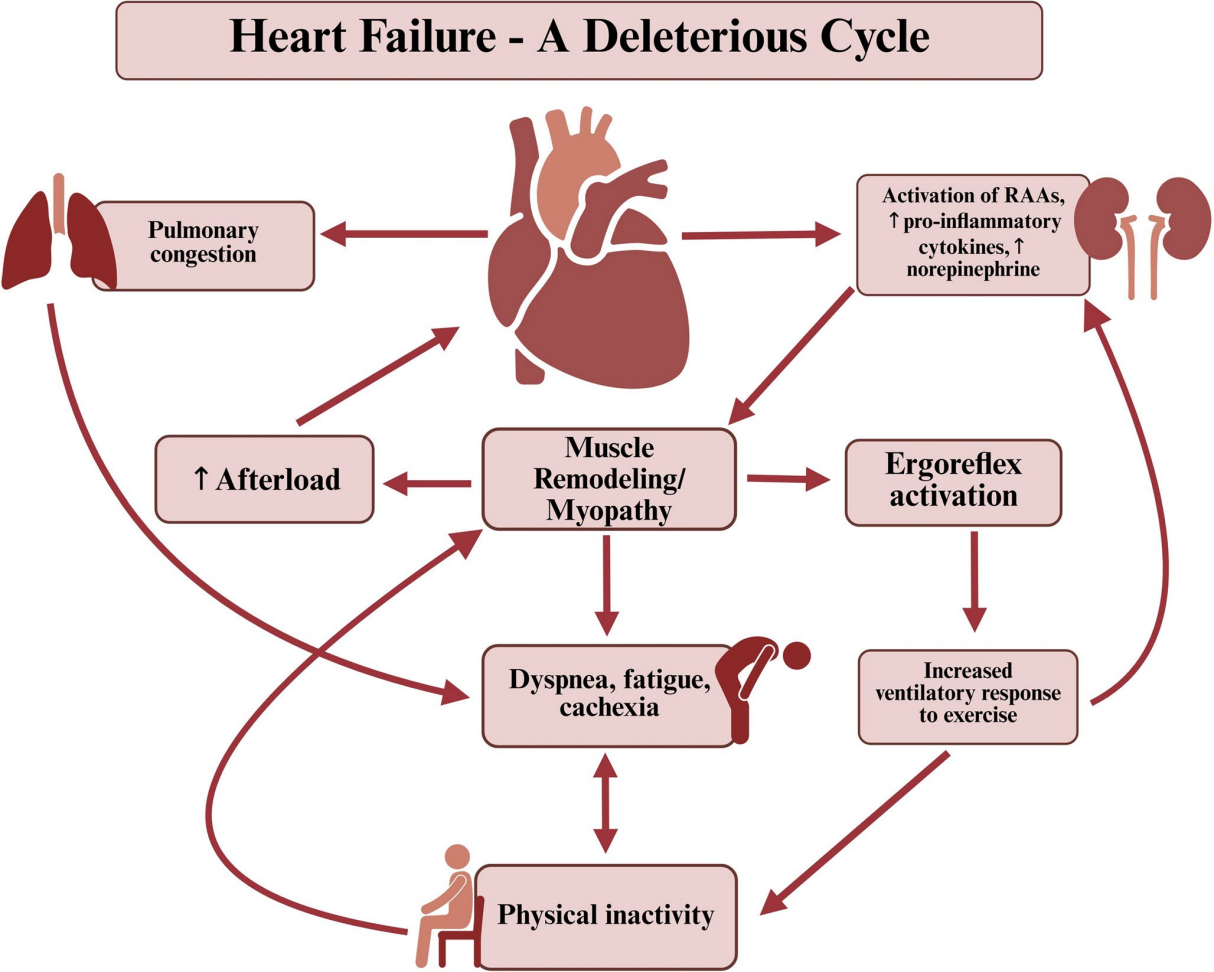
Heart Failure- A Deleterious Cycle.Created in BioRender. Stahl, M. (2025) https://BioRender.com/vdsxhz9

Briefly, the initial LV dysfunction induces several physiological impairments including decreased blood flow and perfusion, physical inactivity and excessive catabolic activity within the skeletal muscle (as well as other system adaptions) [98] (Fig. 2). These changes cumulatively create a more glycolytic profile which induces early fatigue in patients with HF. This promotes further physical inactivity and subsequent muscle atrophy [23, 95]. For example, muscle strength (knee extensor and grip strength) are important predictors of VO_2peak_ in this population and are inversely related to NYHA class [99]. Thus, the syndrome could be viewed as being a vicious cycle, where the dysfunctions of each system result in the further deterioration of others.

This raises the questions of which tissues benefit from exercise rehabilitation in patients with HF and can we design programs to maximize these benefits and increase physical function and health span? In 2012, Haykowsky et al. [30] performed a secondary analysis on a previously conducted study looking at the effect of 16 weeks of aerobic exercise [33] in patients with HFpEF. Following exercise training there was a 2.3 ml/kg/min increase in VO_2peak_ compared to controls. They found that there were no significant differences in LVEDV, LVESV, SV, systemic vascular resistance or any blood pressures. In contrast, they found that 84% of the improvement in cardiorespiratory fitness was attributed to an improved a-vO_2_diff while only 16% were due to cardiac adaptations [30]. Thus, in this analysis, it appears central adaptations may provide negligible improvements for fitness and targeting the skeletal muscle may play a beneficial role in improving the severe exercise intolerance seen in this population.

## Other Modes of Exercise Training in HF

### Resistance Training

Resistance training (RT) is normally employed for conditioning skeletal muscle tissue, however, it was largely overlooked for patients with CHF prior to 1990’s due to concerns that high cardiac afterload may adversely affect left ventricular remodelling [100, 101]. Modern methods of hemodynamic measurement have allayed these concerns by confirming the integrity of the left ventricle during RT [102, 103].

The 2021 Physical Rehabilitation for Older Patients Hospitalized for Heart Failure (REHAB HF) [104] trial demonstrated that in fragile acute decompensated HF patients a physical exercise intervention (functional movements and tasks) was clinically beneficial in comparison to an attention control.

To date relatively few studies have looked at RT in outpatient exercise rehabilitation setting [36, 42, 105, 106]. Two meta-analyses examined the effects of RT as a single intervention versus usual care [107, 108] or aerobic training [108] and found increases in lower and upper body muscle strength, aerobic capacity and QoL, without any detrimental effect on left ventricular parameters. They both concluded that in patients with HF who are unable or not inclined to partake in aerobic activity, RT alone is appropriate to elicit meaningful benefit. and may offer an alternative approach.

### High Intensity Interval Training (HIIT)

When prescribing exercise rehabilitation, it is important to remember the intensity of exercise relative to the patient’s starting fitness. A typical individual with HF has a VO_2peak_ of ~ 13 ml/kg/min (3.7 METS) which classifies activities of daily living such as dressing ~ 7 ml/kg/min (2.5 METS) and vacuuming ~ 10.5 ml/kg/min (3.3 METS) to be approximately 68–89% of their maximal effort [109]. Essentially tasks of daily living are at or above the anerobic threshold (high intensity) for this population, with lactate and H^+^ accumulation a regularly a limiting factor (which requires intermittent rest). Prescribing exercise at in a moderate aerobic “zone” (i.e. ~40–80% of VO_2peak_) is less than they must do daily. It is likely insufficient to maximize the stimulus and elicit exercise adaptations in the skeletal muscle tissues. It may be necessary to perform brief bouts of HIIT to allow patients with HF to stimulate peripheral muscle adaptations.

In patients with HFrEF, the largest trial to date; SMARTEX-HF trial (*n* = 261), found increases in VO_2peak_ with both HIIT, and moderate intensity compared to usual care, but no significant differences between intensities [110]. Those in the HIIT group saw a significant difference in LV end-diastolic diameter compared to usual care that was not seen in the moderate group. Conversely in HFpEF the OptimEx-Clin trial (*n* = 154) showed the largest gain in VO_2peak_ following moderate intensity exercise after 3 months [29].

Two recent meta-analyses (15 and 16 studies) report that HIIT appears to show greater benefit for improving VO_2peak_, LVEF and HRQoL in HF generally [111, 112]. Although when broken down by sub-types there may be a greater benefit for HIIT in patients with HFrEF [111].

HIIT also appears to be safe in patients with other forms of HF outlined in this review. A recent multi-site RCT found in 80 HCM patients randomized equally to 12 weeks of HIIT versus usual care found improvements in exercise tolerance (VO_2peak_ and VO_2_ at the anaerobic threshold), psychological markers of depression and hospital anxiety, and no increased risk for dangerous arrythmias [113]. Similarly, another smaller study (*n* = 15) compared 5-months of moderate intensity versus progressive HIIT [64]. They found improvements in VO_2peak_ regardless of exercise intensity, and though underpowered to evaluate safety, no adverse clinical events were observed [64] (see other cardiomyopathies section for specific guidelines for each variant). Similarly, in ANOCA a feasibility study evaluating 3-months of HIIT resulted in robust improvements in VO_2peak_ and flow-mediated dilation with no unexpected adverse events reported [114].

The current literature appears to unanimously support that HIIT is safe in patients with HF. The data in HCM and ANOCA also support the safe use of HIIT, although the data is less robust and further confirmatory findings in larger cohorts is required [63, 115]. It seems that in HF there may be additional benefits from HIIT training as part of an exercise rehabilitation program (given the very low initial exercise capacities) and patients could benefit from the ability to choose their preferred exercise intensity or implement a mixture of both approaches in a periodized manner.

## New Approaches To Exercise Rehabilitation in HF: Targeting the Peripheral Tissues

**Fig. 3:**
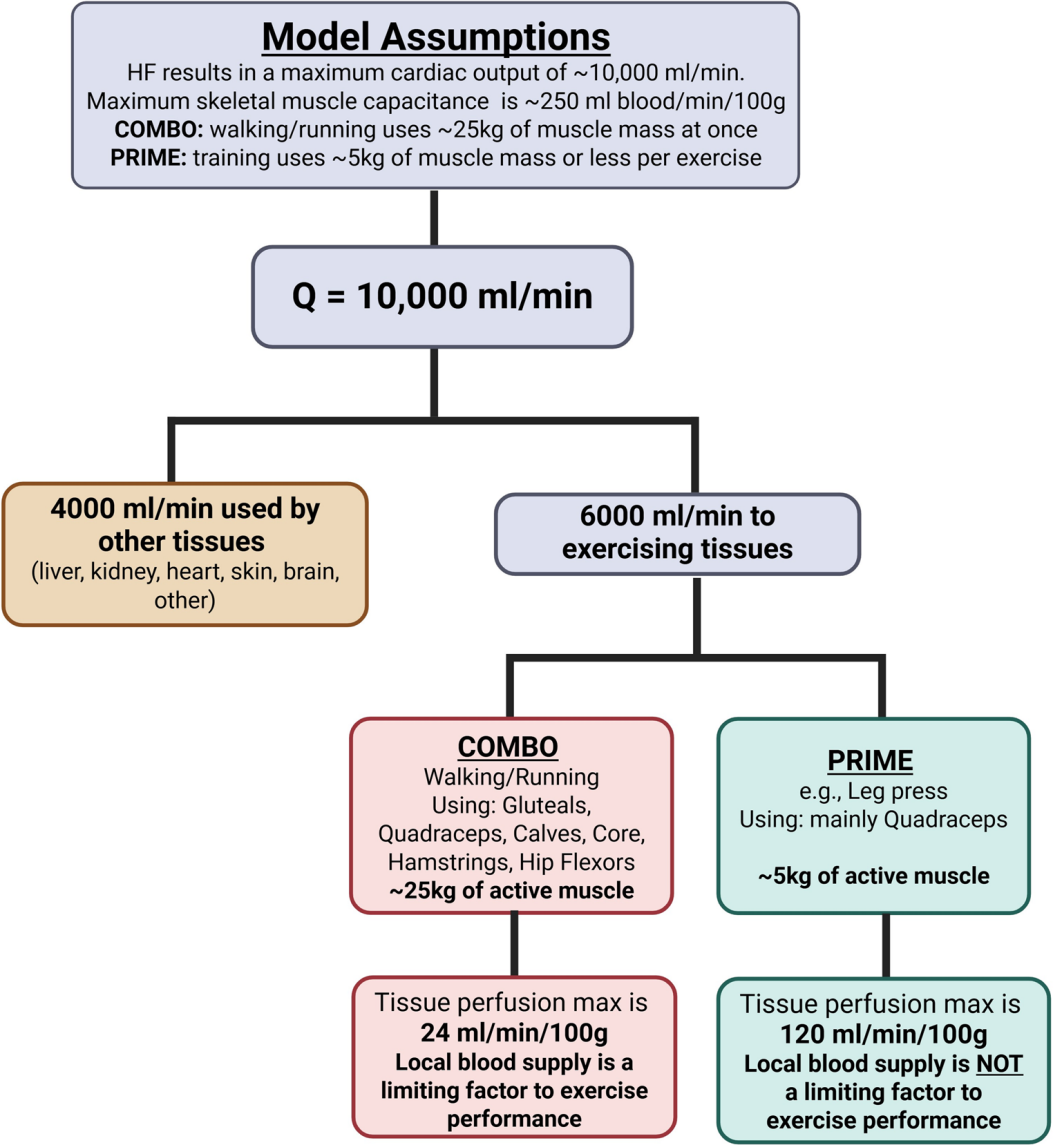
The Theoretical Basis for PRIME in Heart Failure. Created in BioRender. Stahl, M. (2025) https://BioRender.com/7fn5rqd

Based on the hypothesis that physical function in older individuals is predominantly limited by impairments in peripheral tissues, we developed the Peripheral Remodeling via Intermitted Muscular Exercise (PRIME) approach [116]. The theoretical underpinnings of PRIME in HF can be seen in Fig. 3. For traditional whole body-type exercise training approaches (COMBO), skeletal muscle perfusion on a unit basis (~ 24 ml/min/100 g) is a fraction of that theoretically available during PRIME (~ 120 ml/min/100 g) in which muscle groups are used in a more isolated approach. This allows for a ‘hybrid’ aerobic-resistance program, designed to deliver an optimal localized training stimulus to the peripheral vasculature, muscle, and bone without imposing excess cardiovascular strain. In practice, each PRIME exercise involves contractions of specific isolated muscle groups with a moderate load, defined as 40%–50% of their maximal voluntary capacity for a more extended period (up to 5 min). Heart rate is usually below 50–60% of predicted max [117]. PRIME is used for an initial period (~ 4 weeks) to maximize peripheral adaptations prior to more traditional fully body exercise training regimen.

We initially demonstrated greater benefits from the PRIME approach in 76 older individuals (*≥* 70yrs) at risk of losing functional independence (but not HF). Four initial weeks of PRIME (versus AT), followed by 8 weeks of AT and RT, generated significantly greater increases in VO_2peak_, 1RM strength, Senior Fitness Test Scores [117], brachial and aortic blood pressure [118], and bone mineral density [118],

In a follow-up pilot study of 19 older HFrEF patients (*≥* 65 year, mean EF of 31.6%) with a baseline VO_2peak_ of 13.5 ml/kg^/^min [119], performed either PRIME or AT + RT for 4 weeks (twice/week), followed by 4 weeks of COMBO for all participants. The group randomized initially to PRIME significantly increased VO_2peak_, anaerobic threshold, and maximal strength at 8 weeks when compared to baseline. In comparison, the group starting with COMBO only showed a significant increase in strength. No major adverse events occurred during this study. A larger (*n* = 92) trial in this cohort is ongoing (5R01AG75556, NCT05609097).

## Summary/Conclusions

As new data accumulates, the guidelines for exercise for patients with HF and associated cardiomyopathies continue to evolve. Currently, it appears, given appropriate precautions and clinical assessments, exercise is a unifying therapy across most heart failure conditions regardless of ejection fraction (except AVRC). Contrary to the beliefs from as little as 60 years ago, exercise training appears to be safe, and beneficial in terms of improvements in functional capacity and health-related QoL. Larger controlled trials are needed to better examine the impact of exercise on hard clinical endpoints such as HF hospitalizations (likely beneficial) and cardiovascular mortality. There is also growing evidence to support exercise rehabilitation as a cost-effective strategy for preventing HF related events (compared to UC) [44].

As we progress into the era of personalized medicine, the emerging question is not if exercise is beneficial for HF, but what type of exercise may generate the best outcomes in different populations. Given the very low functional capacities of many HF patients, activities of daily living often require metabolic demands above the anaerobic threshold and lactate turn-point and are therefore intermittent by necessity. Our improved understanding of the role of skeletal muscle in the progression of HF suggests, to generate meaningful adaptations in peripheral tissues we may have to incorporate HIIT training (relative to an individual’s abilities) or a form of resistance training into exercise regimens. Our current approach of PRIME evolved as an attempt to find a middle ground whereby the periphery is stimulated to adapt without imposing a high workload on the heart. Other novel approaches should be encouraged.

Two caveats to the current HF and exercise literature are clear; (a) a vast majority of studies are in HF patients much younger than the average age of diagnosis and are predominantly male; and (b) there is still a need there is a need for larger studies to demonstrate the efficacy of exercise training in cardiomyopathies such as HCM and ANOCA. It is therefore unclear that current guidelines are optimized for these populations and research should focus on developing data to address these questions.

## Data Availability

Not applicable.
