# Correction: Exercise Rehabilitation for Heart Failure and Associated Cardiomyopathies

**DOI:** 10.1007/s11886-026-02352-w

**Published:** 2026-03-20

**Authors:** Macy E. Stahl, Nathan R. Weeldreyer, James P. MacNamara, Patricia F. Rodriguez Lozano, Jason D. Allen

**Affiliations:** 1 Department of Kinesiology, School of Education and Human Development, University of Virginia, Charlottesville, VA 22903 USA; 2 Faculty of Nursing, College of Health Sciences, University of Alberta, Edmonton, AB Canada; 3 Department of Radiology and Medical Imaging, University of Virginia Health, Charlottesville, VA USA; 4 Department of Medicine, University of Virginia School of Medicine, Charlottesville, VA 22904 USA

**Correction: Current Cardiology Reports (2025) 27:171**

10.1007/s11886-025-02313-9

In recently published article, the authors have identified some minor corrections required in the main body.

These corrections are typographical in nature and do not affect the results, discussion, or conclusions of the article.

The original article has been corrected.

